# Development and validation of a clinical prediction model for postoperative weight regain after bariatric surgery using inflammatory, metabolic, and ferritin biomarkers

**DOI:** 10.20945/2359-4292-2026-0033

**Published:** 2026-04-01

**Authors:** Yaxin Liu, Chenxi Fu, Longhao Sun

**Affiliations:** 1 Endocrinology and Metabolism Department, Tianjin Medical University General Hospital, Tianjin, China; 2 General Surgery Department, Tianjin Medical University General Hospital, Tianjin, China

**Keywords:** Bariatric surgery, postoperative weight regain, biomarkers, key predictors

## Abstract

**Objective:**

Postoperative weight regain remains a challenge after bariatric surgery and affects long-term outcomes. This study aimed to develop a clinical model to predict weight regain within 12 months, prior to surgery by using preoperative inflammatory, metabolic, and ferritin as biomarkers.

**Subjects and methods:**

This retrospective observational study included 394 patients with obesity who underwent bariatric surgery (2020-2023), including laparoscopic sleeve gastrectomy (LSG) and laparoscopic Roux-en-Y gastric bypass (LRYGB). Patients were divided into a training set (70%, n = 276) and a validation set (30%, n = 118) using a random number table. Weight regain was defined as a ≥ 10% increase from the postoperative nadir (median time to regain: 8.2 months). Key variables included peripheral blood inflammatory markers [systemic immune-inflammation index (SII, calculated as platelet count × neutrophil count/lymphocyte count), neutrophil-to-lymphocyte ratio (NLR)], glycolipid metabolism indicators [low-density lipoprotein cholesterol (LDL-C), high-density lipoprotein cholesterol (HDL-C)], and ferritin levels. Multivariate logistic regression was used to identify independent predictive variables, and the nomogram model was validated via calibration, area under the receiver operating characteristic curve (AUC), and decision curve analysis (DCA).

**Results:**

The weight regain rate was 19.9% (55/276) in the training set. Independent predictive variables included elevated SII (OR=1.004; 95% CI = 1.000-1.007), LDL-C (OR = 1.873; 95% CI = 1.054-3.329), ferritin (OR = 1.005; 95% CI = 1.003-1.008), and reduced HDL-C (OR = 0.103; 95% CI = 0.013-0.844) (all P < 0.05). The model showed strong discrimination (training AUC = 0.852, 95% CI = 0.795-0.910; validation AUC = 0.812, 95% CI = 0.709-0.915) and good calibration (Hosmer-Lemeshow P > 0.05). DCA confirmed the model’s clinical utility across threshold probabilities.

**Conclusion:**

Preoperative SII, LDL-C, ferritin, and HDL-C levels effectively predict postoperative weight regain. Early monitoring of these biomarkers may guide personalized interventions to improve long-term outcomes.

## INTRODUCTION

With the development of the global economy and changes in people’s lifestyles, the incidence of obesity has been increasing rapidly ^(1)^. The World Health Organization reported that in 2022, the number of people with obesity worldwide exceeded 1 billion ^(2,3)^. Obesity not only severely affects an individual’s physical health but also significantly increases the risk of chronic diseases such as cardiovascular diseases, diabetes, hypertension, and fatty liver ^(4,5)^. Bariatric surgery has been widely recognized for its safety and effectiveness after years of development and practice ^(6)^. By changing the structure of the gastrointestinal tract and the digestion process, bariatric surgery can significantly reduce the weight of patients and improve glycolipid metabolism disorders ^(7)^. However, postoperative weight regain is a critical issue affecting the long-term efficacy of bariatric surgery, with reported rates ranging from 15% to 35% within 2-5 years postoperatively ^(8)^.

This study focused on weight regain within 12 months postoperatively (the early stage) with the goal of establishing a predictive tool that does not cover the long-term weight regain cycle (2-5 years) most commonly reported in clinical practice. Postoperative weight regain not only leads to weight rebound in people with obesity but also may trigger the recurrence and progression of metabolic diseases such as diabetes and hypertension, which may cause psychological problems such as depression and anxiety ^(9,10)^.

Currently, research on postoperative weight regain in people with obesity focuses mainly on anatomical factors, diet and exercise habits, gut hormones and psychological status ^(11)^. Some studies have also indicated that after bariatric surgery, the secretion levels of peptides that promote satiety, such as peptide YY, glucagon-like peptide-1, and gastric inhibitory polypeptide, increase, whereas the secretion level of ghrelin, which promotes appetite, decreases ^(12,13)^. In addition, a low exercise frequency and insufficient exercise duration and intensity after bariatric surgery are considered important reasons for postoperative weight regain ^(14)^.

Although many factors have been found to be closely related to postoperative weight regain after bariatric surgery, there is still a clinical need to identify specific biological markers that can predict the risk of postoperative weight regain earlier and with greater objectivity. Previous studies have proposed new ideas for understanding the risk of weight regain from the perspectives of inflammation and glycolipid metabolism disorders ^(15-18)^. Ferritin is the main form of stored iron, but its level is also affected by systemic inflammation. Thus, changes in ferritin levels may reflect both iron metabolism status and inflammatory activity, which together have implications for people with obesity ^(19)^.

Despite the existing research on postoperative weight regain and related indicators in people with obesity, providing clinicians with a precise prediction tool to guide personalized treatment and prevent weight regain is currently not possible. Therefore, this study aimed to construct a model to predict postoperative weight regain before surgery by using preoperative inflammatory markers, glycolipid metabolism indicators, and ferritin levels to enable early risk assessment and personalized interventions.

## SUBJECTS AND METHODS

### Study design and participants

This study was an analytical, observational, open-label retrospective study. The experimental designers, data collectors, and statistical analysts were independent of each other, and the data collectors and statistical analysts were blinded to the experimental design. People with obesity who underwent bariatric surgery in our hospital from January 2020 to January 2023 were selected as the research subjects.

**Inclusion criteria:** ^(1)^ aged 18-65 years; ^(2)^ body mass index (BMI) ≥ 30 kg/m^2^ (WHO criteria); ^(3)^ underwent bariatric surgery [laparoscopic sleeve gastrectomy (LSG) or laparoscopic Roux-en-Y gastric bypass (LRYGB)] at our hospital; ^(4)^ complete preoperative laboratory data (inflammatory markers, glycolipid metabolism, iron metabolism) and postoperative follow-up records (≥ 12 months); and ^(5)^ voluntarily signed informed consent.

**The exclusion criteria were as follows:** ^(1)^ patients who had malignant tumors or hematological/autoimmune diseases affecting inflammation or iron metabolism; ^(2)^ patients who used drugs to modify inflammation, glycolipid metabolism, or iron metabolism within 3 months preoperatively; ^(3)^ patients affected by mental/cognitive impairment that made it impossible to cooperate with follow-up; ^(4)^ pregnant or lactating women; and ^(5)^ patients who had incomplete clinical or follow-up data.

**Sample size:** In accordance with previous studies on postoperative weight regain in people with obesity ^(20)^, the expected weight regain rate of enrolled patients after bariatric surgery was 20%. The confidence interval was set at 95%, and the allowable error was 5%. The following sample size estimation formula was used: *n*=*Z*^2^×*p*×(1-*p*)/*E*^2^, where *n* is the sample size, *Z* = 1.96, the expected pass rate (*p*) = 0.20, and the allowable error (*E*) = 0.05. Substituting into the above formula, *n*≈245.86. Rounding up and considering that 10% of cases might be unable to be included in the analysis, at least 274 people with obesity who underwent bariatric surgery needed to be included. A total of 394 eligible people with obesity were ultimately included in this study.

**Figure 1:**
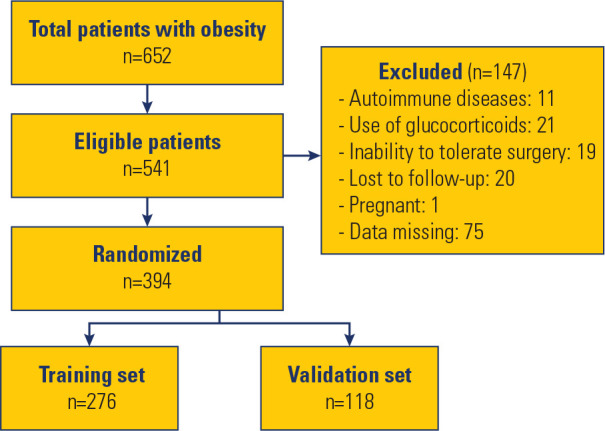
Flowchart of the enrollment of research subjects.

**Division of sets and groups:** The enrolled patients were divided into a training set (*n* = 276) and a validation set (*n* = 118) at a 7:3 ratio using the random number table method. A patient enrollment flowchart is shown in **Figure 1**. All enrolled patients were evaluated for postoperative weight regain according to the following criteria: weight gain ≥ 10% compared with the lowest postoperative weight within 12 months after bariatric surgery was defined as weight regain. Patients with weight regain were defined as the weight regain group, and patients who did not meet the above criteria were defined as the nonweight regain group. In this study, only a retrospective statistical analysis of the previous inpatient database was conducted, and the patient names and contact information were kept confidential. Accordingly, the ethics committee waived any specific ethical requirements for this study.

### Data collection and outcome measures

#### General clinical data

The general clinical data of the enrolled patients, including age, sex, preoperative BMI, waist-hip ratio, length of hospital stay, operation time, surgical approach, obesity type, and comorbidity status, were collected. The obesity types were divided into abdominal obesity and generalized obesity. The surgical approaches included mainly LSG and laparoscopic Roux-en-Y gastric bypass. The comorbidities included mainly type 2 diabetes mellitus (T2DM), fatty liver, obstructive sleep apnea hypopnea syndrome (OSAHS), hypertension, hyperlipidemia, hyperuricemia, and thyroid nodules.

#### Peripheral blood composite inflammatory markers

Preoperative fasting peripheral venous blood was collected. The neutrophil count (N), lymphocyte count (L), platelet count (PLT), and monocyte count (MONO) were measured. The derived indices included the neutrophil-to-lymphocyte ratio (NLR) = N/L, the platelet-to-lymphocyte ratio (PLR) = PLT/L, the monocyte-to-lymphocyte ratio (MLR) = MONO/L, the systemic immune-inflammation index (SII) = PLT × N/L, and the systemic inflammatory response index (SIRI) = (MONO × N)/L.

#### Detection of glycolipid metabolism indicators

Preoperative fasting blood was used to measure the levels of glycated hemoglobin (HbA1c), insulin, C-peptide, total cholesterol (TC), triglyceride (TG), high-density lipoprotein cholesterol (HDL-C), and low-density lipoprotein cholesterol (LDL-C).

#### Detection of iron metabolism indicators

Preoperative fasting blood was used to measure serum iron, ferritin, total iron-binding capacity (TIBC), and unsaturated iron-binding capacity (UIBC, calculated as TIBC minus serum iron). Transferrin saturation (TSAT), a key indicator of iron utilization, was further calculated as (serum iron/TIBC) × 100% to complement the iron status assessment.

### Statistical analysis

SPSS 26.0 software and R 4.2.1 software were used for statistical analysis. For measurement data, a normality test was first performed. Data conforming to a normal distribution are expressed as the mean ± standard deviation (*x*¯±*s*), and comparisons between two groups were performed using an independent-samples t test. Measurement data that did not conform to a normal distribution are expressed as medians (interquartile ranges) [M (Q1, Q3)], and comparisons between two groups were performed using the Mann-Whitney U test. Count data are expressed as the number of cases (percentage) [n (%)], and comparisons between groups were performed using the χ^2^ test. When the theoretical frequency was less than 5, Fisher’s exact probability method was used. Factors with P < 0.05 in the univariate analysis were included in the multivariate logistic regression analysis, and the forward stepwise regression method was used to determine the variables independently associated with postoperative weight regain. On the basis of the results of the multivariate logistic regression analysis, the rms package of R software was used to construct a nomogram prediction model. In the training set, the bootstrap resampling method (1000 repeated samples) was used for internal validation of the model, and in the validation set, external validation of the model was performed. Calibration curves were plotted to observe the consistency between the predicted probability and the actual probability; decision analysis curves (DCAs) were plotted to analyze the net benefit of the model at different threshold probabilities; and receiver operating characteristic (ROC) curves were plotted, and the area under the curve (AUC) was calculated to evaluate the predictive efficacy of the model. The larger the AUC is, the stronger the predictive ability of the model. P < 0.05 was considered to indicate statistical significance.

## RESULTS

### Comparison of clinical data between the training set and the validation set

There were no significant differences in the general clinical data, such as age, sex, preoperative BMI, disease duration, obesity type, surgical approach, or comorbidity status, between the training set and the validation set (P > 0.05; **Table 1**), indicating that the clinical data of the patients in the two datasets were comparable and could be used for subsequent research and validation.

**Table 1:** Comparison of general clinical data between the training set and the validation set [(*x*±*s*), *M* (*Q*_1_, *Q*_3_), n (%)]

General clinical data	Training set (n = 276)	Validation set (n = 118)	t/Z/χ^2^	P
Age (years)	31.87 ± 7.45	31.59 ± 7.69	0.334	0.739
Sex			0.648	0.421
Male	80 (29.0)	39 (33.1)		
Female	196 (71.0)	79 (66.9)		
Preoperative BMI (kg/m^2)^	40.32 ± 6.24	39.88 ± 5.50	0.655	0.513
Waist-hip ratio	0.95 (0.90, 1.00)	0.94 (0.89, 1.00)	-0.626	0.532
Number of hospitalization days (d)	6 ^(5,6)^	6 ^(5,6)^	-0.811	0.417
Operation time (min)	90 (75, 105)	90 (70, 100)	-1.070	0.285
Type of obesity			0.329	0.566
Abdominal obesity	141 (51.1)	64 (54.2)		
Generalized obesity	135 (48.9)	54 (45.8)		
Surgical approach			0.937	0.333
LSG	135 (48.9)	64 (54.2)		
LRYGB	141 (51.1)	54 (45.8)		
Comorbidities				
T2DM	108 (39.1)	40 (33.9)	0.965	0.326
Fatty liver	266 (96.4)	115 (97.5)	0.059	0.809
OSAHS	209 (75.7)	90 (76.3)	0.013	0.908
Hypertension	93 (33.7)	33 (28.0)	1.247	0.264
Hyperlipidemia	162 (58.7)	79 (66.9)	2.371	0.124
Hyperuricemia	126 (45.7)	45 (38.1)	1.901	0.168
Thyroid nodule	121 (43.8)	60 (50.8)	1.634	0.201

Note: BMI: body mass index; T2DM: type 2 diabetes mellitus; OSAHS: obstructive sleep apnea hypopnea syndrome.

### Comparison of observed indicators between the weight regain group and the nonweight regain group in the training set

In the training set, 55 patients experienced weight regain within 12 months of surgery, with a weight regain rate of 19.9%. These patients were all included in the weight regain group, and the other 221 patients were included in the nonweight regain group. The prevalences of T2DM, hypertension, and hyperlipidemia and the levels of NLR, PLR, SII, SIRI, HbA1c, C-peptide, LDL-C, and ferritin were greater in the weight regain group than in the nonweight regain group, while the level of HDL-C was lower (P < 0.05). There were no significant differences in age, sex, preoperative BMI, waist-hip ratio, length of hospital stay, operation time, obesity type, surgical type, prevalence of fatty liver, OSAHS, hyperuricemia, thyroid nodules, MLR, insulin, TC, TG, serum iron, TIBC, or UIBC between the two groups (P > 0.05; **Table 2**).

**Table 2:** Comparison of the observed indicators between the weight regain group and the nonweight regain group in the training set [(*x*±*s*), *M* (*Q*_1_, *Q*_3_), n (%)]

Index	Weight regain group (n = 55)	Nonweight regain group (n = 221)	t/Z/χ^2^	P
General clinical data				
Age (years)	32.53 ± 6.66	31.71 ± 7.64	0.731	0.465
Sex			0.955	0.328
Male	13 (23.6)	67 (30.3)		
Female	42 (76.4)	154 (69.7)		
Preoperative BMI (kg/m^2)^	41.05 ± 6.17	40.14 ± 6.26	0.970	0.333
Waist-hip ratio	0.96 (0.91, 1.02)	0.95 (0.90, 1.00)	-0.856	0.392
Number of hospitalization days (d)	6 ^(6,6)^	6 ^(5,6)^	-0.720	0.471
Operation time (min)	90 (75, 110)	90 (70, 105)	-0.144	0.886
Type of obesity			2.362	0.124
Abdominal obesity	23 (41.8)	118 (53.4)		
Generalized obesity	32 (58.2)	103 (46.6)		
Surgical approach			0.765	0.382
LSG	24 (43.6)	111 (50.2)		
LRYGB	31 (56.4)	110 (49.9)		
Comorbidities				
T2DM	29 (52.7)	79 (35.7)	5.331	0.021
Fatty liver	54 (98.2)	212 (95.9)	0.641	0.423
OSAHS	42 (76.4)	167 (75.6)	0.015	0.902
Hypertension	27 (49.1)	66 (29.9)	7.287	0.007
Hyperlipidemia	40 (72.7)	122 (55.2)	5.578	0.018
Hyperuricemia	23 (41.8)	103 (46.6)	0.407	0.524
Thyroid nodule	18 (32.7)	103 (46.6)	3.446	0.063
NLR	2.38 ± 0.71	1.74 ± 0.66	6.316	<0.001
PLR	142.32 ± 36.95	115.49 ± 34.08	5.138	<0.001
MLR	0.24 ± 0.07	0.21 ± 0.11	1.639	0.102
SII	764.71 ± 252.04	498.89 ± 232.07	7.470	<0.001
SIRI	1.24 (0.96,1.54)	0.79 (0.59, 1.09)	5.955	<0.001
HbA1c (%)	7.03 ± 2.05	6.49 ± 1.24	2.509	0.013
Insulin (mU/L)	20.80 (13.60, 26.40)	21.70 (16.35, 30.45)	-1.255	0.209
C-peptide (ng/mL)	4.51 ± 1.95	3.99 ± 1.48	2.176	0.030
TC (mmol/L)	4.93 ± 0.87	4.98 ± 0.92	-0.402	0.688
TG (mmol/L)	1.72 (1.29, 2.68)	1.84 (1.43, 2.61)	-1.037	0.300
HDL-C (mmol/L)	1.01 ± 0.21	1.09 ± 0.19	-2.549	0.011
LDL-C (mmol/L)	3.54 ± 0.83	3.18 ± 0.65	3.390	0.001
Serum iron (µmol/L)	14.05 ± 5.46	14.61 ± 5.34	-0.700	0.484
TIBC (µmol/L)	66.76 ± 10.7	68.14 ± 12.92	-0.728	0.467
UIBC (µmol/L)	54.40 (42.80, 61.40)	52.20 (42.00, 61.85)	0.167	0.867
Ferritin (ng/mL)	146.05 (51.44, 446.11)	94.67 (46.80, 188.95)	2.831	0.005

Note: BMI: body mass index; T2DM: type 2 diabetes mellitus; OSAHS: obstructive sleep apnea hypopnea syndrome; NLR: neutrophil-to-lymphocyte ratio; PLR: platelet-to-lymphocyte ratio; MLR: monocyte-to-lymphocyte ratio; SII: systemic immune-inflammation index; SIRI: systemic immune response index; HbA1c: glycated hemoglobin; TC: total cholesterol; TG: triglyceride; HDL-C: high-density lipoprotein cholesterol; LDL-C: low-density lipoprotein cholesterol; TIBC: total iron binding capacity; UIBC: unsaturated iron binding capacity.

### Risk factors for postoperative weight regain

The postoperative weight regain status of people with obesity was assigned as the dependent variable (no = 0, yes = 1), and the intergroup differential indicators, including T2DM, hypertension, hyperlipidemia, NLR, PLR, SII, SIRI, HbA1c, C-peptide, HDL-C, LDL-C and ferritin (all original values), were assigned as independent variables. Multivariate logistic regression analysis revealed that higher SII values [OR (95% CI) = 1.004 (1.000-1.007)], LDL-C levels [OR (95% CI) = 1.873 (1.054-3.329)], and ferritin levels [OR (95% CI) = 1.005 (1.003-1.008)] were risk factors for postoperative weight regain, and a higher level of HDL-C [OR (95% CI) = 0.103 (0.013-0.844)] was a protective factor (P < 0.05; **Table 3**).

**Table 3:** Multivariate logistic regression analysis of the risk of postoperative weight regain in people with obesity

Index	β	S.E.	Wald χ^2^	P	OR	Lower limit of 95% CI	Upper limit of 95% CI
T2DM	0.201	0.455	0.194	0.660	1.222	0.501	2.983
Hypertension	0.414	0.422	0.962	0.327	1.512	0.662	3.455
Hyperlipidemia	0.099	0.413	0.058	0.810	1.104	0.491	2.482
NLR	0.378	0.589	0.412	0.521	1.460	0.460	4.633
PLR	0.008	0.008	1.046	0.306	1.008	0.992	1.024
SII	0.004	0.002	4.503	0.034	1.004	1.000	1.007
SIRI	-0.539	0.672	0.645	0.422	0.583	0.156	2.175
HbA1c	0.148	0.148	1.013	0.314	1.160	0.869	1.549
C-peptide	0.101	0.125	0.660	0.417	1.107	0.866	1.414
HDL-C	-2.273	1.073	4.487	0.034	0.103	0.013	0.844
LDL-C	0.628	0.293	4.572	0.033	1.873	1.054	3.329
Ferritin	0.005	0.001	15.068	0.000	1.005	1.003	1.008
Constant	-7.192	1.967	13.366	0.000			

### Nomogram model for the risk of postoperative weight regain

**Figure 2:**
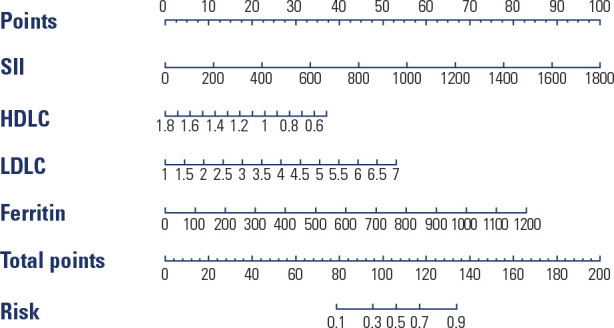
Nomogram model for the risk of postoperative weight regain.

On the basis of the results of the multivariate logistic regression analysis, a nomogram model for the risk of postoperative weight regain was constructed using the SII, LDL-C level, ferritin level, and HDL-C level, as shown in **Figure 2**. It can be intuitively seen from the nomogram that the higher the preoperative levels of SII, LDL-C, and ferritin and the lower the level of HDL-C are, the higher the score and the probability of postoperative weight regain.

### Goodness of fit of the nomogram model for the risk of postoperative weight regain

**Figure 3:**
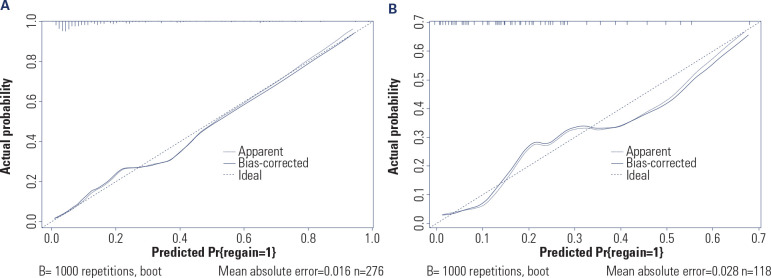
(**A**) Calibration curve analysis of the nomogram model for the risk of postoperative weight regain in the training set; (**B**) Calibration curve analysis of the nomogram model for the risk of postoperative weight regain in the validation set.

The goodness-of-fit of the nomogram model for the risk of postoperative weight regain was evaluated by calibration curves in the training set and the validation set. The results showed that in the training set, the Hosmer-Lemeshow χ^2^ of the model was 7.7129 (P = 0.462), while in the validation set, the Hosmer-Lemeshow χ^2^ of the model was 12.577 (P = 0.127), indicating that the model had a high goodness-of-fit in both the training set and the validation set (see **Figure 3**).

### Predictive efficacy of the nomogram model for the risk of postoperative weight regain

**Figure 4:**
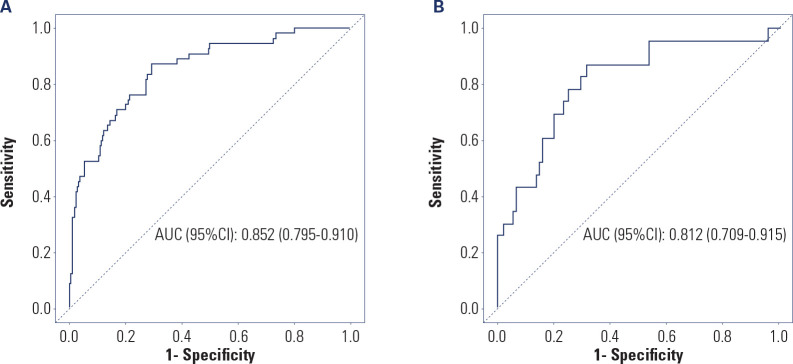
(**A**) ROC curve analysis of the nomogram model for the risk of postoperative weight regain in the training set; (**B**) ROC curve analysis of the nomogram model for the risk of postoperative weight regain in the validation set.

The predictive efficacy of the nomogram model for the risk of postoperative weight regain was analyzed by ROC curves in the training set and the validation set. The results showed that in the training set, the AUC (95% CI) of the model was 0.852 (0.795-0.910); in the validation set, the AUC (95% CI) of the model was 0.812 (0.709-0.915), indicating that the model had high predictive efficacy in both the training set and the validation set (see **Figure 4**).

### Clinical value of the nomogram model for the risk of postoperative weight regain

**Figure 5:**
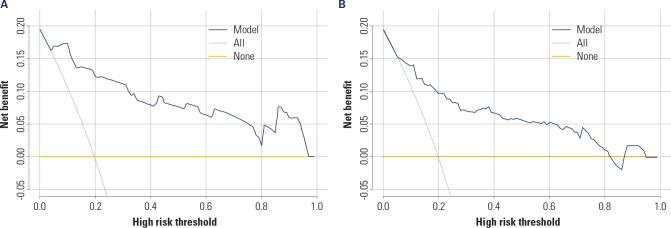
(**A**) DCA curve analysis of the nomogram model for the risk of postoperative weight regain in the training set; (**B**) DCA curve analysis of the nomogram model for the risk of postoperative weight regain in the validation set.

The clinical net benefit of the nomogram model for the risk of postoperative weight regain was analyzed through DCA in the training set and the validation set. The results revealed that in the training set, the clinical net benefit of the model was greater than 0 within a threshold probability range of 0-0.95. In the validation set, the clinical net benefit of the model was greater than 0 within a threshold probability range of 0-0.82. These results suggested that the model had high clinical application value in both the training set and the validation set (as shown in **Figure 5**).

## DISCUSSION

This retrospective analysis of 394 people with obesity who underwent bariatric surgery revealed that prior to surgery, higher levels of LDL-C and ferritin, a greater SII value, and a lower level of HDL-C, were significantly associated with postoperative weight regain. The nomogram prediction model constructed on the basis of the results of multivariate logistic regression analysis demonstrated excellent goodness-of-fit, good predictive efficacy, and high clinical application value through internal and external validation. These findings suggest that the establishment of this model can provide a powerful tool for clinicians to accurately assess the risk of weight regain within 12 months postoperatively at an early stage, facilitating timely adjustment of short-term intervention strategies. Notably, the model cannot support claims about durable weight control, as the 12-month follow-up period is insufficient to reflect long-term outcomes.

The results of this study indicated that the SII value in the weight regain group was significantly greater than that in the nonweight regain group, and multivariate logistic regression analysis revealed that a higher SII value was associated with an increased risk of postoperative weight regain. Previous studies have explored the role of the SII in predicting the outcomes of cancer patients and found that a high SII value is related to a poor prognosis ^(21)^. A chronic inflammatory state may disrupt fat metabolism and energy balance, thereby increasing the risk of postoperative weight regain ^(22)^. Although differences in the NLR and PLR were observed between the weight regain group and the nonweight regain group in this study, they did not show independent predictive value in the multivariate analysis. Some studies have suggested that the NLR can reflect the body’s inflammatory response and immune status and that an elevated NLR in people with obesity may be associated with adipose tissue inflammation and metabolic disorders ^(23)^. The inflammatory response affects fat metabolism and weight regulation through multiple pathways, leading to postoperative weight regain ^(24)^. Inflammatory factors can interfere with the insulin signaling pathway, resulting in insulin resistance, which promotes fat synthesis and storage while inhibiting fat breakdown, ultimately leading to weight gain ^(25,26)^.

This study also revealed that a higher LDL-C level was a risk factor for postoperative weight regain, whereas a higher HDL-C level was a protective factor, which is consistent with the general understanding of lipid metabolism abnormalities in people with obesity. An elevated LDL-C level reflects abnormal lipid metabolism in the body and may be related to postoperative weight regain ^(27)^. In contrast, HDL-C has an anti-atherosclerotic effect. It can transport cholesterol from peripheral tissues back to the liver for metabolism, reducing cholesterol deposition in the vascular wall ^(28)^. A decrease in the HDL-C level may indicate a decrease in the body’s ability to clear lipids, thereby increasing the risk of weight regain ^(29)^. In this study, although differences in the HbA1c level, C-peptide level, and LDL-C level were observed between the weight regain group and the nonweight regain group, only the LDL-C level showed independent predictive value in the multivariate analysis. Some studies have suggested that HbA1c can reflect long-term blood glucose control and that abnormal blood glucose metabolism is closely related to the development of obesity ^(30,31)^. The differences in the research results may be related to factors such as the baseline characteristics of the study subjects, surgical methods, postoperative follow-up duration, and lifestyle interventions. In people with obesity, a decrease in the HDL-C level may be related to reduced HDL synthesis, increased HDL degradation, and impaired liver function ^(32,33)^. Previous studies have also suggested that inflammatory factors can modify HDL, causing it to lose its normal antioxidant and anti-inflammatory functions and rendering it unable to effectively perform reverse cholesterol transport ^(34,35)^.

This study is the first to investigate the ferritin level as a factor influencing postoperative weight regain in people with obesity and found that an elevated ferritin level is a risk factor for postoperative weight regain. Ferritin is the main form of stored iron, but it is also an acute-phase reactant - it is closely regulated by chronic low-grade inflammation, which is a core feature of obesity. In people with obesity, inflammatory cytokines can upregulate hepatic ferritin synthesis, whereas elevated ferritin further exacerbates inflammation by promoting oxidative stress in adipocytes ^(36-38)^. This bidirectional interaction jointly disrupts energy metabolism: iron overload impairs fat decomposition via lipid peroxidation ^(39)^, and inflammation disrupts insulin signaling and energy balance ^(25)^, ultimately increasing the risk of postoperative weight regain. Ferritin levels in children with obesity are positively correlated with indicators such as the BMI and body fat percentage ^(40)^. Changes in the level of ferritin, which is the main form of stored iron, are closely related to energy metabolism and fat accumulation ^(41,42)^. Iron overload can increase lipid peroxidation in adipocytes, disrupting cell membrane structure and function and thus affecting fat synthesis and metabolism ^(38,39)^. Iron overload may also interfere with the signal transduction pathway in adipocytes, affecting the expression of genes related to fat metabolism by upregulating the gene expression of fatty acid synthase (FAS) in adipocytes and promoting fatty acid synthesis while inhibiting the activity of enzymes related to fat breakdown, resulting in fat accumulation ^(43,44)^. In people with obesity, iron overload may lead to energy metabolism imbalance, decreased fat oxidation, and increased fat synthesis, thus promoting weight gain. Changes in the expression of genes related to energy metabolism in the adipose tissue of people with obesity may be related to changes in ferritin levels ^(45,46)^.

The nomogram prediction model constructed in this study is highly important for clinical prediction and assessment. This model incorporates preoperative measures of SII, LDL-C, ferritin, and HDL-C, providing clinicians with an intuitive, convenient, and accurate tool for assessing the risk of postoperative weight regain in people with obesity considering bariatric surgery. For patients with higher SII, LDL-C, and ferritin levels and lower HDL-C levels, doctors can ascertain that they have a greater risk of postoperative weight regain. For such patients, doctors can strengthen preoperative health education, enabling patients to fully understand the risk of postoperative weight regain and the importance of a healthy lifestyle. After surgery, doctors can more closely monitor patients at risk in regard to weight changes, metabolic indicators, and lifestyles; promptly detect signs of weight regain; and take corresponding intervention measures. This may include increasing the frequency of follow-up and regularly measuring patients’ blood glucose, blood lipids, and inflammatory markers to adjust the treatment plan in a timely manner. Compared with the previous lack of effective prediction tools, where doctors could rely only on experience and some simple clinical indicators to predict the patient’s risk of weight regain, the prediction model in this study has greater accuracy and reliability.

Some limitations of this study should be considered. First the sample size remains insufficient compared with that of large-scale multicenter studies. In research on risk factors for postoperative weight regain in patients with obesity, an inadequate sample size may result in the failure to detect some weak yet real associations, thereby affecting the accuracy and reliability of the results. Second, this study defines weight regain as an increase of ≥ 10% from the lowest weight within 12 months after surgery. While this enables the early identification of potential risks, clinically significant weight regain mostly occurs 2-5 years postoperatively. Therefore, the predictive efficacy of this model for weight regain beyond 1 year after surgery still needs to be verified. Third, ferritin is an acute-phase reactant and cannot be used alone as a marker of iron stores in people with obesity, as its level is confounded by both iron metabolism and systemic inflammation. Although we calculated TSAT to improve the iron utilization assessment, this study lacked data on C-reactive protein and hepcidin levels. This limitation prevents us from distinguishing whether elevated ferritin reflects iron overload or inflammatory activation, weakening the mechanistic interpretation of ferritin’s role. Fourth, considering that obesity is a complex multifactorial disease, postoperative weight regain may be influenced by many other factors, such as the gut microbiota, genetic polymorphisms, and psychological factors.

To address these limitations, multicenter, large-scale studies should be conducted in the future, encompassing patients with obesity from different regions and age groups, to increase the diversity and representativeness of the sample. Moreover, the follow-up duration should be extended to 2-5 years to more accurately distinguish between early fluctuations and persistent regain, further improving the external validity of the model. In addition to focusing on peripheral blood composite inflammatory markers, glycolipid metabolism disorders, and ferritin characteristics, indicators such as the gut microbiota, gene polymorphisms, and psychological factors should be incorporated into the research. Through a comprehensive analysis of the relationships between these indicators and postoperative weight regain, the mechanism underlying postoperative weight regain can be elucidated more deeply, and a more robust prediction model can be established.

In conclusion, the findings of this study suggest that higher preoperative levels of SII, LDL-C, and ferritin and a lower level of HDL-C in people with obesity are important factors for predicting the risk of postoperative weight regain. Timely monitoring of these markers can help predict the risk of postoperative weight regain at an early stage, providing ideas for improving patients’ surgical prognosis, adjusting postoperative treatment strategies in a timely manner, and enhancing the clinical treatment effect.

## Data Availability

the datasets used and/or analyzed during the current study available from the corresponding author on reasonable request.
